# Ecological niche overlap in the Arctic vegetation influenced by seabirds

**DOI:** 10.1038/s41598-023-30809-3

**Published:** 2023-03-16

**Authors:** Adrian Zwolicki, Katarzyna Zmudczyńska-Skarbek, Agata Weydmann-Zwolicka, Lech Stempniewicz

**Affiliations:** 1 Department of Vertebrate Ecology and Zoology, University of Gdańsk, Wita Stwosza 59, 80-308 Gdańsk, Poland; 2 Institute of Oceanography, University of Gdańsk, Piłsudskiego 46, 81-378 Gdynia, Poland

**Keywords:** Community ecology, Ecological modelling, Ecosystem ecology

## Abstract

In the High Arctic, nutrients are the most limiting resources, so terrestrial vegetation is of low complexity and grows slowly. However, locally, large seabird colonies increase soil fertility by deposition of faeces, supporting the development of rich and fast-growing plant communities. Here, we assessed how seabird colonies affected ecological niche segregation of plants, across the fertilisation gradient. Study sites were located near five little auk colonies, distributed longitudinally across the Svalbard archipelago. We described vascular plant composition and identified 13 environmental variables, based on which, we calculated and tested the niche overlap (NO) between the 18 most frequent species. Based on the hierarchical classification of the NO matrix, we distinguished typical High Arctic Vegetation (HAV), and Bird-Cliff Vegetation (BCV). The BCV was characterised by higher average NO and soil *δ*^15^N compared to HAV. The highest NO values across the fertilisation gradient were found on the border between the distinguished communities and were positively correlated with species diversity. We suggest that in the High Arctic, seabirds-delivered nutrients lead to the development of separate plant communities through the mechanism of avoiding inter-species competition, while simultaneous high species diversity and NO are related to high facilitation between plants on the border between the communities.

## Introduction

Climate change in the Arctic causes rapid environmental shifts, including higher temperatures, precipitation, humidity and streamflow, as well as reduction in snow-cover, what significantly influences the function of its terrestrial ecosystems^1^. High Arctic terrestrial ecosystems are especially vulnerable because they are relatively simple in terms of structure and functioning, mostly resulting from extremely low air temperatures, low rate of organic matter decomposition, and nutrient limitations^2–4^. However, the Arctic consists of a mosaic of different landscapes, where poor environments frequently neighbour relatively nutrient-rich habitats, overgrown with highly productive vegetation, which are observed in the vicinity of large seabird colonies, mostly as the result of their fertilisation^5,6^. Such strong environmental gradients between these barren and lush habitats create a perfect opportunity for investigating the coexistence of plant species within a community, ecological niche differentiation, and the distribution of species across niche space. This is especially important in the light of recent changes observed in terrestrial and marine environments, which jointly affect Arctic colonial seabirds^7^, and consequently also impact terrestrial vegetation^5,6^.

The most numerous and widespread High Arctic colonial seabirds are little auks (dovekies, *Alle alle*), which globally reach ca. 37 million pairs, of which approximately 1 million pairs breed on the Svalbard archipelago^8,9^. During the breeding season, they form large colonies and act as biovectors between two ecosystems; marine, where they forage, and terrestrial, where they deposit nutrients, mostly in the form of droppings^5,10^. In this way little auks determine the development of terrestrial vegetation, either by enhancing plant abundance or locally influencing plant community composition^6,11^. Little auks are planktivorous, and in the Svalbard archipelago they feed mainly on large copepods present in the waters of the Arctic origin, and avoid Atlantic water that contains smaller copepods^12^; however, the progressing warming in the Arctic and connected shifts in water masses distribution significantly influence the numbers and structure of seabird community^7^, with little auks being especially prone to them. Given the important role of large seabird colonies in shaping the Arctic tundra vegetation, it is crucial to learn to what extent seabirds modify relationships between plants, and how it affects vegetation niche segregation, in order to predict how climate change will affect Arctic vegetation communities.

The common measure of relationships between species within a community is niche overlap (NO), which is useful in describing species interactions and ecological specialisation in response to environmental conditions^13–15^. We based our study on the Hutchinsonian definition of an ecological niche, which describes the *n-*dimensional hypervolume of resources required for species’ long-term survival^16,17^. The whole hypervolume, defined by *n*-environmental variables, represents a fundamental, or a potential niche, within which a particular species often occupies its smaller portion (mostly due to competitive exclusion), defined as a realised niche. Because species often rely on many resources, such niche definition is consistent also with the Grinnellian definition^18^. In terms of the hierarchical organisation of plant diversity, a realised niche corresponds to a local community scale (*α* niche, *α* diversity), in which interactions among species could be observed^19^. In the most basic approach, niche breadth (range of values within an environmental gradient) and position (the niche optimum) describes the response of a species along an environmental/resource gradient^20,21^.

Niche overlap refers to a situation in where co-occurring species share parts of their niche space. Initially, the niche overlap between two species was often interpreted as a measure of their competition for limited resources, as competition theory states that community structure may be shaped by the partitioning of resources between coexisting species^22^. Therefore, quantifying the degree of resource partitioning is a key component of studies examining community structure and species coexistence. Generally, field experiments have revealed that only three or four resources are limiting for any plant community^23^. The partitioning of a few key nutrient resources has been proposed to explain the coexistence of different plant species within a given community^24^. In the High Arctic tundra, one of the most limiting factors is nutrient availability, and in the case of soil nitrogen concentration, the dominant plant species uses its most available form^4,25^. So, nutrients, subsidised by colonial seabirds, could act as a strong environmental factor modifying relationships between High Arctic terrestrial plants and also be responsible for niche segregation between species, and consequently influencing the formation of vegetation communities.

Our study aimed to describe changes in *α* realised niche overlap (NO) of vascular plant species across the gradient of ornithogenic nutrient enrichment in High Arctic plant communities, and to assess the importance of seabird nutrient enrichment for the segregation of plant niches. Simple and natural Arctic terrestrial ecosystem, with extremely strong influence of colonial seabirds on otherwise generally poor Arctic vegetation, is a perfect place to study relationships between plants. To calculate NO, we used a statistical method proposed by Geange^26^, testing if selected pairs of species occupied the same niche, and if all vascular plant species were distributed in clusters across a niche space. We hypothesise that average niche overlap within a plant community would be distributed unimodally, with the highest niche overlap located on the border between nutrient-poor habitats, overgrown by stress-resistant species, and highly fertilised habitats, occupied by competitive species.

## Methods

### Ethical statement

Tissue collection was undertaken on non-endangered plant species which had no significant impact on the plant population at the site. The study complies with the Guidelines for researchers in Svalbard required by the Governor of Svalbard and was performed under Governor of Svalbard permission nos. 2007/00150-2 a.512, 2007/00150-5 and 2007/00150-9.

### Study area

**Figure 1:**
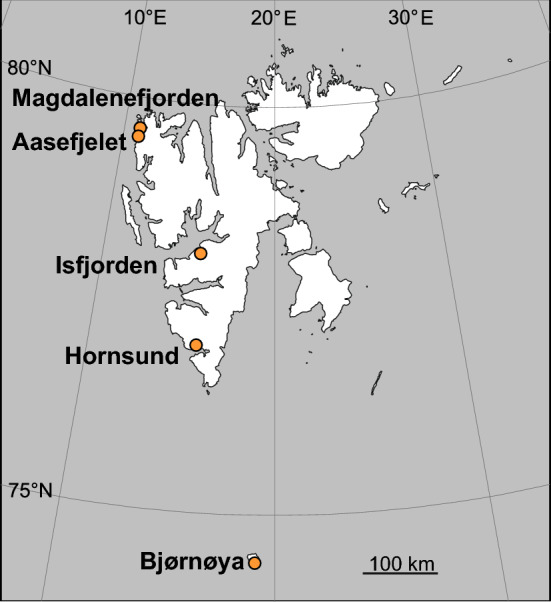
Distribution of five study locations within the Svalbard archipelago (The map was created with the use of CorelDRAW 2019).

The study was conducted in the vicinity of five little auk colonies distributed longitudinally across the Svalbard archipelago (Fig. 1), in areas of different local climate and oceanographic regimes:

1. Magdalenefjorden (north-west Spitsbergen, 79.58°N 11.03°E). Three sampling transects were located within the fjord: two on the slopes of Høystakken and one on Skarpegga, all descending to the sea. There was a large breeding colony of little auks, numbering ~ 18,000 pairs. The fjord is influenced by relatively warm Atlantic water masses carried by the West Spitsbergen Current, with a periodic influx of colder polar waters from the Arctic Ocean^27^.
2. Aasefjelet (north-west Spitsbergen, 79.52°N 10.70°E). Two study transects were defined along the slope, both adjacent to a large little auk colony (~ 36,000 pairs), and descending to the open sea. Local climatic conditions are like those in Magdalenefjorden, except that the area is directly exposed to the open sea.
3. Isfjorden (central part of west Spitsbergen, 78.24°N 15.34°E). Two transects were situated along the western slope of Platåberget, descending to the sea, close to a small little auk colony (~ 250 pairs) in Bjørndalen. This is the warmest part of Svalbard due to the regular significant inflow of warm Atlantic water masses^27^.
4. Hornsund (a fjord in south-west Spitsbergen, 77.01°N 15.51°E). One sampling transect was located along the gentle slope of Ariekammen, below a large colony of little auks (~ 23,500 pairs). Another transect was situated along the southern slope of Fugleberget with the same exposure, ca. 1 km distant, and with the distinctly low influence of the colony. Local climatic conditions in the fjord are influenced mostly by the Sørkapp Current that flows around the southern tip of Spitsbergen, carrying Arctic water masses from the north-western part of the Barents Sea to the north, and occasionally by the inflows of warmer Atlantic waters from the West Spitsbergen Current^7,28^.
5. Bjørnøya (a small, highly exposed island midway between Spitsbergen and northern Norway, 74.38°N 19.03°E). One transect site was situated on a gentle slope of Alfredfjellet, exposed to the north and descending to the shore of Ellasjøen (a lake), close to a medium-sized colony of little auks (~ 10,000 pairs). The second site was in parallel but ca. 500 m distant from the colony and separated from it by a temporary stream. Bjørnøya is surrounded to the east, south and west by the polar front, i.e. the area where cold Arctic water from the north and east is mixed with warm and stratified Atlantic water and has a typical maritime climate^29^.

### Sampling

Data were collected in July and August, during six expeditions to: Hornsund (2005 and 2007), Magdalenefjorden and Aasefjelet (2007 and 2009), Bjørnøya (2008), and Isfjorden (2010) We established 12 line transects in the vicinity of the five colony locations. The number of sampling plots (160 × 160 cm each) within each transect depended on the local geomorphological conditions, and ranged from 5 to 12 plots: Magdalenefjorden 3 transects (24 sampling plots), Aasefjelet 2 (18), Isfjorden 2 (18), Hornsund 3 (26 plots), Bjornoya 2 (10); in total 97 plots. The starting point of each transect was located close to the colony, then it run down the slope and ended on the seashore. Plots were distributed as follows: plot 1 (starting point), plot 2 (6 m), 3 (15 m), 4 (29 m), 5 (49 m), 6 (79 m), 7 (125 m), 8 (193 m), 9 (296 m), 10 (449 m), 11 (680 m), and 12 (1026 m)^5^.

### Plant species composition

Within each sampling plot, we visually estimated the percentage cover of previously identified vascular plant species (in total 36 species), the contribution of moss cover to the total vegetation cover, and calculated the Shannon (H’) alpha diversity index^30^. To calculate realized niche overlap values (NO) within the vegetation community, we selected species observed at least in eight sampling plots to meet the minimum sample size requirements. Consequently, 18 plant species were used in further analyses.

### Physical and chemical soil properties

The physicochemical analyses were performed from three soil samples collected from three sites along the same diagonal of each plot, to describe the importance of key abiotic factors for vascular plants development and niche utilisation. Each sample was taken with a shovel from the soil surface layer (to a depth of ca. 5–10 cm) and contained approximately 500 cm^3^ of soil. In the field laboratory or at the University Centre in Svalbard (UNIS), and no later than 24 h after the collection, each sample was split into three subsamples, each containing 80 cm^3^ of soil, which were used to assess:

(i) soil moisture (%): Soil subsamples were weighed with an electronic scale (precision 0.1 g) before and after oven-drying (60 °C) to a constant mass. Soil moisture was defined as soil moisture = [(wet mass – dry mass) dry mass^–1^] 100%.
(ii) soil conductivity (*μ*S cm^−1^) and pH: A fresh soil subsample was mixed with 160 cm^3^ of distilled water. The mixture was shaken for ca. 20 min and then filtered through a sieve (0.5 mm mesh). Conductivity and pH were quantified in the filtrate using a pH/conductivity/salinity meter CPC-401 (Elmetron).
(iii) Nitrate (NO_3_^−^), ammonium (NH_4_^+^), potassium (K^+^) and phosphate (PO_4_^3−^) content (mg 1000 g^−1^ soil dry mass): Soil subsamples of 80 cm^3^ were mixed with 200 cm^3^ 0.03 N acetic acid and left for ca. 60 min while being shaken regularly. The solution was then filtered through a sieve (0.5 mm mesh) and filter paper (MN 640 w, Macherey–Nagel Φ = 125 mm). The filtrate was analyzed using a photometer LF205 following standard procedure^31^.

### Stable isotope analyses

To assess whether seabird nutrient enrichment was important for plants niche segregation, we used stable nitrogen (*δ*^15^N) and stable carbon (*δ*^13^C) signatures, and the percent of total C and total N in the soil. *δ*^15^N and *δ*^13^C signatures were previously reliably documented as a good proxy of marine-derived ornithogenic nutrient supplies^6,32–34^. Although the evaluation of stable isotopes, together with the total amounts of elements, are measurements generated routinely during a stable isotope analysis, in our study it was important to track how the levels of fertility were dependent on bird fertilisation.

To evaluate *δ*^15^N and *δ*^13^C signatures we used soil subsamples, which were previously used to calculate soil moisture. Soil was sieved (0.25 mm mesh) to remove stones and large plant debris, and grounded with a vibrating mill (LMW-S, Testchem) to a grain size of less than 0.03 mm. Before isotopic analyses, we removed inorganic carbon (by adding 1 mL HCl 1N per 100 mg of soil) and lipids (using 4 ml of cyclohexane per 50 mg of soil). Afterwards, a small amount of each subsample (1–2 mg, weighed with a microbalance, precision 0.001 mg) was packed into a tin capsule.

Nitrogen and carbon isotope ratios were determined by a continuous flow mass spectrometer (Thermo Fisher, Delta V Advantage) coupled to an elemental analyser (Thermo Fisher, Flash EA 1112). All samples for stable isotopes were analysed in one laboratory at the University of La Rochelle (France). Results were expressed in the conventional *δ*^15^N and *δ*^13^C notation, according to the equation: *δ* X = (*R*_*sample*_* R*_*standard*_^−1^ – 1) 1000 (‰), where *R*_*sample*_ was the stable isotope ratio ^15^N/^14^N or ^13^C/^12^C in the analysed sample, and *R*_*standard*_ was the stable isotope ratio ^15^N/^14^N or ^13^C/^12^C (respectively) in the reference material i.e. atmospheric N_2_ for nitrogen and PeeDee belemnite for carbon^35^.

### Statistical analyses and data management

To include plant species abundances in the niche overlap measurements, we disaggregated the 18 most frequent species into 40 pseudo-species, based on five cut-levels (cl) of their percentage ground cover within each sampling plot (the idea borrowed from the TWINSPAN method^36,37^). The first cut-level (cl1) describes the entire range of species abundance starting from 0.1 to 100% of its ground cover, cl2 describes the abundance of species starting from 2 to 100%, cl3: from 5 to 100%, cl4: from 10 to 100%, and cl5: from 20 to 100% ground cover. Therefore, a single species (e.g., *Cerastium arcticum*) could be divided maximally into five pseudo-species corresponding to their cut-levels.

The NO values (ranging between 0 and 1, where NO is 0 when the two distributions are completely disjoint, and 1 when they exactly coincide) were calculated for each pair of 40 pseudo-species over niche axes using method described in Geange et al.^26^. The unified analysis of NO allows multiple niche axes to be analysed, even if they represent different data types, which would otherwise require different statistical treatments^26^, e.g., in our study: soil physical and chemical properties (continuous variables), and the relative abundance of mosses (continuous percentage) in the sampling plots. NO was calculated using two types of niche axes: (i) for the multivariate niche space calculations, 12 measured environmental variables were reduced to 3 orthogonal axes using Principal Component Analysis (PCA) to avoid influence of significant correlation between variables; and (ii) for each environmental variable as a niche axis, separately. Niche overlap, based on nonparametric kernel density functions (NO_*K*_) on axis *t*, was calculated as:

$${\mathrm{NO}}_{{K}_{i;j;t}}=1-\frac{1}{2}\int |{f}_{it}\left(x\right)-{f}_{jt}(x)|dx$$

where *f*_*it*_ and *f*_*jt*_ are the kernel population density functions for species *i* and *j*^26^.

We ran two separate suites of NO analyses, the first one focusing exclusively on the 18 most common species to test the species distribution across niche space, and the second one focusing on the obtained 40 pseudo-species. For each suite of analyses, we used appropriate transformations and probability models (as described in Geange et al.^26^) to calculate NO between species/pseudo-species *i* and *j* for each data type, and obtained a unified measure of niche overlap by averaging NO between species/pseudo-species *i* and *j* over each different niche axis. We then used null model tests, as described in Geange et al.^26^, to determine (i) if the relative position of species/pseudo-species pairs in niche space differed; and (ii) if multiple species/pseudo-species were clustered within a niche space. All the raw niche overlap results are presented in the Supplementary Information 1.

Because 1 – NO may be viewed as a measure of distance between the pairs of species, we constructed *a n *×* n* distance matrix and used non-metric multidimensional scaling (nMDS) to display niche similarities between all pseudo-species, and hierarchical classification with the unweighted pair-group method with arithmetic mean (UPGMA) to identify groups of species/communities that occupied similar niche space. The relationship between all niche axes was described using PCA and correlation analyses, which are presented in the Supplementary Information 2.

The relative importance of the studied environmental variables for the plant niche segregation was described as an average NO matrix calculated for each variable separately. Afterwards, the mean NO values between all environmental variables were compared using the Anova Welch test (unequal variances) and the Games–Howell test for pairwise comparisons. Pearson correlations were performed to test the relation between *δ*^15^N and total nitrogen in the soil.

NO, n-MDS, *t-test*, Welch test with *post-hoc* Games-Howell test, correlations, and graphical presentation of the data were performed in R^38^ and the following packages: tidiverse^39^, ggridges^40^, rstatix^41^. NO analyses were conducted using the R code from the method proposed by Geange et al. in^26^. The PCA and Shannon H’ were performed using Canoco 5.0^42^.

## Results

### Species distribution across niche space

**Figure 2:**
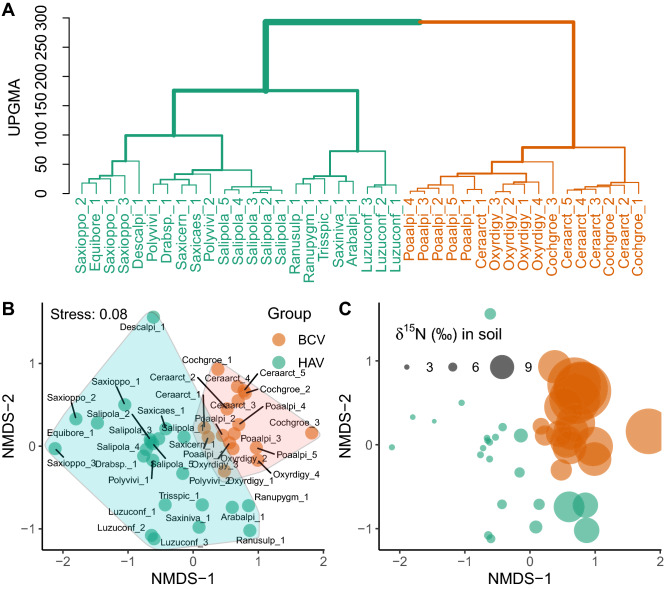
Intra- and interspecific similarities in average niche overlap (NO) between 18 vascular plant species (divided into 40 pseudo-species) calculated for 3 niche axes presented as the results of hierarchical clustering (agglomeration method: UPGMA) (**A**); and non-metric multidimensional scaling (NMDS) (**B**, eclipses represent clusters of pseudo-species with 60% similarity levels; and **C**, the size of a point describes soil *δ*^15^N level). *HAV* High Arctic vegetation, *BCV* bird-cliff vegetation. Species names abbreviations were based on the first four letters of the Latin genus and species names, e.g., *Arabis alpina* = Arabalpi; while the following numbers refering to cut-off levels of pseudo-species.

The vascular plants distribution across the whole niche space was significantly clustered (*p* < 0.001). UPGMA hierarchical classification performed on values of 1 − NO matrix between all pairs of species revealed two main distinct clusters (Fig. 2A, Supplementary Table S1a–c). Similarly, n-MDS illustration of the 1 − NO matrix clearly showed the clumped species distribution across niche space, and each of the two distinct groups included species that occupied similar niches (Fig. 2B,C). The first group consisted of species typical for ornitocoprophilous communities, known as bird-cliff vegetation (BCV), of which the most abundant were: *Cerastium arcticum*, *Cochlearia groenladica*, *Poa alpina*, and *Oxyria digyna*. Some species from BCV occupied niche spaces that were not identified as significantly different, according to a null model test (e.g., *C. arcticum*_1 and *O. digyna*_1; NO = 0.80, SD = 0.174, *p* = 0.103). The second group consisted of the common High Arctic tundra (HAV) species e.g., *Salix polaris, Saxifraga oppositifolia, Luzula confusa*, and *Polygonum viviparum*, whose niches also did not differ significantly (e.g. *S. oppositifolia*_1 and *S. polaris*_1; NO = 0.80, SD = 0.163, *p* = 0.148; Fig. 2, Supplementary Table S1a–c). This grouping of species within a niche space was caused by the three, out of twelve studied soil parameters: *δ*^15^N (*p* < 0.001), *δ*^13^C (*p* = 0.001), and PO_4_^3^ (*p* = 0.018) content in the soil, which was also confirmed by the significant clustering results during the analysis of species distribution of each environmental variable separately.

Clusterisation across niche space was the result of the high variability of niche overlap values between plant species. The largest NO was observed between *C. arcticum_*1 and *P. alpina*_1 (two pseudo-species with 1st cut-levels within the BCV community), both occupying niche spaces that were not different (NO = 0.93, SD = 0.115, *p* = 0.978). On the contrary, the most separated were the species of the 3rd cut-levels: *C. groenladica_*3 from BCV and *S. oppositifolia_*3 from HAV (NO = 0.31, SD = 0.20, *p* < 0.001; Fig. 2B Supplementary Table S1a–c). The average niche overlap within the BCV community was higher compared to HAV (0.68 and 0.63, respectively; t-test, *t* = 6.21, *p* < 0.001). All the intra- and interspecific NO similarities between the selected pairs of species were presented in Supplementary Table S1a.

### The importance of nutrients derived by seabirds for vascular plants niche segregation

**Figure 3:**
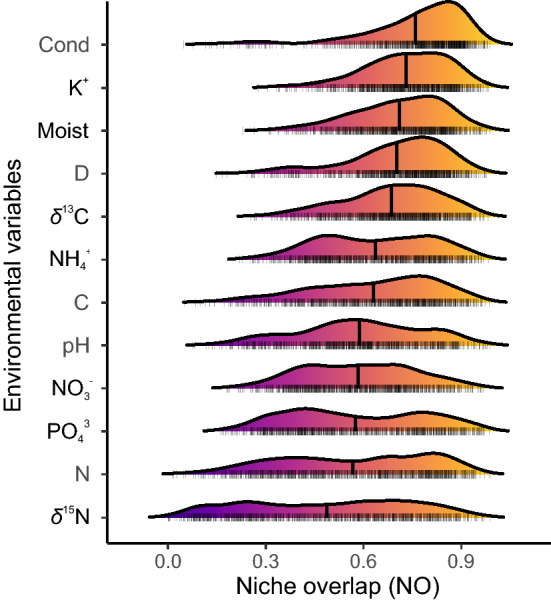
Density plots of niche overlap values (NO) between all plant species for each of the studied environmental variables. The order of the *y*-axis was sorted ascending by average niche overlap, presented as a vertical quantile line. *Cond* conductivity of soil solution, *Moist* soil moisture, *D* moss and lichen ground cover, *C* total carbon in the soil, *N* total nitrogen. The detailed *post-hoc* comparisons of average NO between all variables are presented in Supplementary Table S2.

Seabird nutrient enrichment described by the soil *δ*^15^N signature was the most important variable for plants niche segregation, and showed the lowest average NO between pseudo-species (0.49) and differed significantly from all the remaining environmental variables (Welch Anova: *F* = 144.73, *df* = 3678.5, *p* < 0.001; Games-Howell tests: all *p* < 0.005, see Supplementary Table S2), and had the highest variance (Fig. 3). It was followed by the total soil N and PO_4_^3−^ (both average NO = 0.57), NO_3_^−^ (0.58), soil pH (0.59), total C (0.63), NH_4_^+^ (0.64), and *δ*^13^C (0.68). The remaining four variables (D, soil moisture, K^+^, and conductivity) had relatively low importance for the niche segregation, with average NO ranging from 0.70 to 0.76.

**Figure 4:**
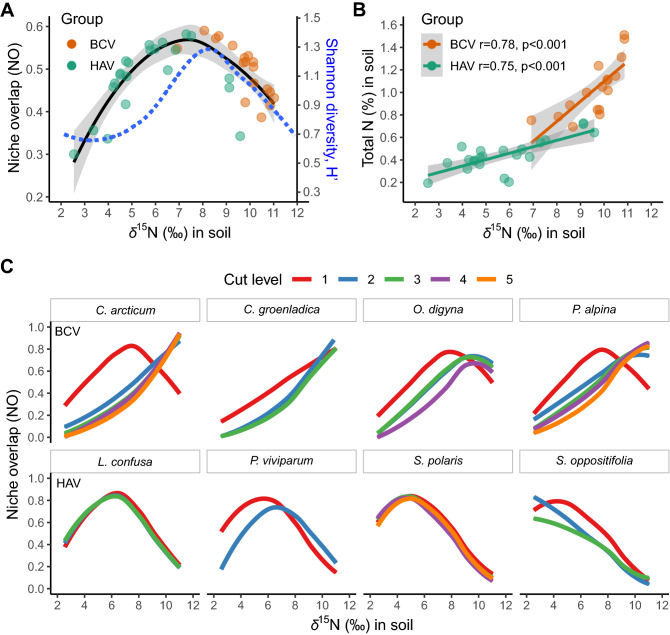
Average niche overlap values (NO) to the nitrogen stable isotope signature (*δ*^15^N) in soil, calculated for 18 vascular plant species divided into 40 pseudo-species, with the Shannon diversity index (blue line) (**A**). Relationships between *δ*^15^N content and the percentage of total nitrogen (N) (**B**). Niche overlap (NO) responses of the eight most abundant plant species to *δ*^15^N gradient, presented as loess regression lines separately for each of their cut-level. Upper panel: bird-cliff vegetation (BCV), lower panel: High Arctic vegetation (HAV) (**C**).

Since seabird nutrient enrichment was the most important factor differentiating the ecological niche of vascular plants, therefore the relation between niche overlap values of each studied pseudo-species was calculated exclusively for soil *δ*^15^N signature and presented across the gradient of soil *δ*^15^N. This relationship was unimodal with the maximum average NO = 0.59 found for *C. arcticum*_1. This NO peak was located around soil *δ*^15^N = 8‰; therefore, this value was recognised as the border between the two previously distinguished plant communities, HAV and BCV (Fig. 4A). Moreover, the distribution of average NO showed a convergent course and similar position of the peak as the Shannon diversity index (H’). The graphs also show a clear distinction between the niche positions of HAV and BCV species, in which species belonging to BCV occupied niches with a significantly higher signature of *δ*^15^N (permutated t-test, *t*_*p*_ = 7.89, *p* = 0.002), and higher total nitrogen (*t*_*p*_ = 9.08, *p* = 0.002), than HAV species (Fig. 4A).

Together with the increasing ratio of nitrogen stable isotope in soil (the average values calculated for each pseudo-species separately), the percentage of total soil N increased significantly (BCV: *r* = 0.78, *p* < 0.001, while HAV: *r* = 0.75, *p* < 0.001), although in the case of bird-cliff vegetation the increase was more rapid (Fig. 4B).

The relationships of NO for the eight most abundant plant species (the four most representative ones for each vegetation community) with distinction to their cut-levels, changed along the *δ*^15^N gradient (Fig. 4C). In general, in the case of species from the bird-cliff vegetation community, the NO increased with *δ*^15^N, although a clear linear increase was visible only for *C. groenlandica* (all its cut-levels) and all the other BCV pseudo-species from the highest abundance levels (cl3-cl5 cut-levels). On the contrary, in the case of species forming HAV, their niche overlap generally decreased with soil *δ*^15^N. The highest NO values amongst BCV species were observed above 8‰ of *δ*^15^N, especially in the case of *C. groenlandica,* while in the case of HAV, below 8‰ of* δ*^15^N axis, e.g., ~ 4‰ of *δ*^15^N in *S. polaris,* and around 6‰ in *L. confusa*. Moreover, pseudo-species from the highest cut-levels, which represented a relatively high abundance of a given species within a community, showed higher niche separation compared to that from their 1^st^ cut levels (Fig. 4C)*.*

## Discussion

Seabirds profoundly impact the surroundings of their nesting sites, mainly through faeces deposition, which creates nutrient-rich habitats occupied by ornitocoprophilous plant species, distinctly differing in composition from the tundra vegetation sensu stricto^6,43,44^. Our study shows that the development of distinct plant communities, defined as bird-cliff vegetation (BCV) and High Arctic vegetation (HAV), located at the opposite ends of the seabirds-fertilisation gradient resulted from the strong vascular plant’s niche segregation as a response to allochthonous nutrients delivered by seabirds. Moreover, the higher level of soil fertility in the bird-cliff vegetation community resulted in a higher average niche overlap between plant species, compared to the High Arctic vegetation. However, the largest niche overlap across the gradient of seabird’s fertilisation was found in the ecotone between the communities, which increased species diversity probably due to facilitation between plants.

### Niche overlap analysis reveals separate plant communities across the gradient of ornithogenic nutrient enrichment

Classification based on niche overlap matrix between species revealed that the studied vascular plants were significantly clustered across the whole niche space and formed two quite distinct communities composed of species with relatively similar niche positions within each community which represent communities with different life strategies. The bird-cliff vegetation comprised exclusively of ornitocoprohilous, short-living and fast-growing species such as *P. alpina*, *C. arcticum*, *C. groenladica*, and *O. digyna*^11^. While in the typical High Arctic tundra community, consisted of species well adapted to low nutrient levels, such as the herbaceous *L. confusa* and *P. viviparum,* and long-lived and slowly growing woody subshrubs, such as *S. polaris*, *S. oppositifolia*, *S. ceaspitosa*, and *S. nivalis*^43^.

Typically in polar regions, areas with no seabird influence are at best covered with low-productive tundra, predominated with dwarf shrubs and/or mosses^45–47^. Usually, vegetation response to intense seabird fertilisation is manifested by the domination of fast-growing graminoids, which in our study were represented by *P. alpina*^45,48,49^. Our results suggest that the development of BCV communities was primarily driven by the competitive advantage of ornitocoprofilous vascular plants that were able to respond rapidly to increased nutrient levels^50,51^. Moreover, faster nitrogen uptake promoted also plants other than grasses, characterised by high growth rates, rapid tissue turnover, and relatively low nutrient use efficiency, exemplified by *C. groenlandica*, *C. arcticum*, and *O. digyna*^52^. Such attributes allowed them to outcompete the slow-growing species, i.e. *S. oppositifolia* or *S. polaris* with long-lived leaves and high nutrient use efficiency^53^. This also corresponds with the theory that species are organised in communities and maintain the presence of separate assemblies to reduce competition, which in turn has a stabilising effect on ecosystems^50^. Moreover, when the context of continuum versus discontinuum controversy is considered, our study suggests that strong environmental gradients, such as the subsidy of marine-origin nitrogen by seabirds, lead to almost complete replacement of typical tundra vegetation by the ornithocoprophilous plant species, and consequently move towards the discontinuum model^54^.

Classic niche theory states that competition between organisms occurs due to resource limitation^23^. Many studies have concluded that competition for limited nutrients is one of the most important factors determining the distribution of plant species^14,25,55,56^. Our results confirmed that the vegetation community from nutrient-reach habitats, like BCV, had higher mean niche overlap compared to nutrient-poor High Arctic vegetation. This larger niche separation in HAV is commonly understood as the mechanism of competition avoidance^23,57^.

Some of the studied plant species inhabited similar niche spaces, especially within a particular community e.g. *C. arcticum*_1 and *O. digyna*_1 in BCV, or *S. oppositifolia*_1 and *S. polaris*_1 in HAV, which seems to be contradictory to the ecological niche theory, which assumes that two species cannot occupy the same niche^17^. The reason for this result could be (i) an insufficient number of niche axes included in the analyses, and (ii) that plants are not only competing for consumable resources but also for inconsumable ones, like physical soil space or light^58–60^, and/or (iii) that coexisting species use various forms of nutrients^50^. Such resource partitioning was reported previously in a nitrogen-limited Alaskan tussock tundra as an important mechanism allowing plant co-occurrence within a community^25^.

### Importance of seabird nutrient enrichment for the segregation of plants ecological niches

Soil stable nitrogen isotope became previously reliably documented as a representation of marine-derived ornithogenic nutrient supplies^6,32–34^. In our study the gradient of soil *δ*^15^N had a fundamental meaning for niche differentiation, showing the lowest average niche overlap values and highest NO variability compared to other tested soil parameters and was responsible for species clusterisation across the niche space^54,61^. The above confirms the key role of seabirds in shaping Arctic plant communities through nutrient enrichment^6^.

McKane’s ^15^N-tracer field experiment revealed that resource partitioning was responsible for the use of the most available nitrogen forms like nitrate by dominant species, while less productive species took less abundant forms, like glycine or ammonium. Here, we compared the importance of four soil nitrogen measurements: *δ*^15^N, total N, NO_3_^−^ and NH_4_^+^. The result was that soil stable nitrogen isotope made the highest contribution to niche differentiation and was followed by the total N and nitrate, while ammonium was the least important. Therefore, we suggest that the comparison of nitrogen ionic forms included in our study showed that nitrates were the most available nitrogen form, which is a similar finding to that for the Alaskan tussock tundra^25^.

The average niche overlap of each pseudo-species across the seabird fertilisation gradient showed a unimodal distribution, with the peak at *δ*^15^N of ca. 8‰. This threshold point was not accidental and coincides with the isotopic signature of little auk faeces (8.1 ± 0.5‰) previously reported from one of our study locations, the Hornsund fjord^62^. Moreover, the distribution of average NO and diversity were also similar, which leads to the conclusion that the highest NO values could have resulted from facilitation, which is a diversity-promoting interaction that occurs between stressful and nutrient-rich habitats leading to the formation of the species-rich transition zone between communities^63^. This result corresponds with the currently observed, and discussed, discrepancies in the stress-gradient hypothesis (SGH), where facilitation may be less important at the extreme ends of the gradient^64,65^.

Our results seem contradictory to Tilman’s hypothesis that the competition across fertilization gradients is constant^66^; when analysing the average niche overlap separately within each community, opposite relationships could be observed: in a nutrient-poor HAV community, average NO increased with increasing nutrient levels; however, in BCV, there was an inverse correlation. These observations suggest that competition may decrease with increasing nutrient subsidies but only in stressed nutrient-limited environments. From the opposite end of the gradient, in highly fertilised part of BCV, the stress level was probably also high, therefore in this case we observed low NO values of the whole plant community and low species diversity.

Niche overlap of the eight most abundant plant species, calculated across the seabird nutrient enrichment gradient, showed how each species shared its niche with other species, and where the NO of a particular species reached the highest values. It was seen that in the case of BCV, NO reached its highest values above* δ*^15^N 8‰, and in the case of HAV below 8‰. This relation was unimodal in the case of the first pseudo-species (which described all occurrences of a species regardless of its quantity), but in pseudo-species of higher abundances, it was more linear. Consequently, if species were more abundant, they occupied different niche positions, causing greater niche separation. In addition, the average niche overlap was generally the highest for the first cut-level (which described the whole range of species abundances) and gradually decreased with subsequent cut levels. Conceivably, highly abundant species were not burdened by interspecific competition, but the intraspecific competition was density-dependent and may have grown along with increasing species abundance^67^. Intraspecific competition could potentially be more intense than the interspecific competition because organisms of the same species had the greatest overlap in niche space, and used the same source/form of nutrients^23,68^. When nutrient availability was sufficient, and plants competed only for space, a winning strategy could have been to dominate habitat space/cover, and this is why we observed only a few highly abundant species at the end of the gradient in the BCV community^57^.

## Conclusions

1. The most important environmental factor that caused plant species niche segregation were marine-origin nutrients supplied by seabirds, described by the *δ*^15^N signature in the soil. This factor caused the studied vascular plants to cluster across the whole ecological niche space, resulting in the formation of two distinct plant communities typical for the bird-cliff (BVC) and High Arctic (HAV) vegetation. The development of these separate communities, which consisted of species characterised by different life strategies and nutrient requirements, was probably caused by the mechanism of inter-species competition avoidance.
2. Along the seabird fertilisation gradient, the average niche overlap of the whole plant community had a unimodal distribution, similarly to the plant species diversity. The highest niche overlap values were found on the border between the BCV and HAV communities, where the *δ*^15^N signature was around 8‰, which had been previously described as the signature of little auk faeces. The greater species diversity and niche overlap in the transition zone between the distinguished plant communities was most likely promoted due to the facilitation between plants.
3. Average niche overlap between plants decreased with increasing species abundance, which was especially visible in the case of dominant species from the bird cliff vegetation. This suggests that the magnitude of intraspecific competition may increase, while the magnitude of interspecific competition decreases, with increasing species dominance within a community.

## Supplementary Information

Supplementary Table S1.

Supplementary Table S2.

Supplementary Table S3.

Supplementary Information 1.

Supplementary Information 2.

## Data Availability

The row and generated datasets that support the findings of this study were provided in Supplementary Table S3 and Supplementary Table S1a–c.
