# Supplementary material for: Ecological niche overlap in the Arctic vegetation influenced by seabirds
Source: Sci Rep. 2023 Mar 16;13:4405. doi: 10.1038/s41598-023-30809-3 (PMC10020437; doi:10.1038/s41598-023-30809-3)
Supplement: Supplementary file 5 — Supplementary Information 2. [file 41598_2023_30809_MOESM5_ESM.pdf]

Ecological niche overlap in the Arctic vegetation influenced by seabirds

Adrian Zwolicki1[*], https://orcid.org/0000-0003-2710-681X

Katarzyna Zmudczyńska-Skarbek[1], https://orcid.org/0000-0003-2276-4565

Agata Weydmann-Zwolicka[2], https://orcid.org/0000-0002-6655-6613

Lech Stempniewicz[1], https://orcid.org/0000-0001-9405-7320

[1] University of Gdańsk, Dept. of Vertebrate Ecology and Zoology, Wita Stwosza 59, 80-308 Gdańsk, Poland

[2] University of Gdańsk, Faculty of Oceanography and Geography, Piłsudskiego 46, 81-378 Gdynia, Poland

[*]Corresponding author:

E-mail: adrian.zwolicki@ug.edu.pl,

PCA

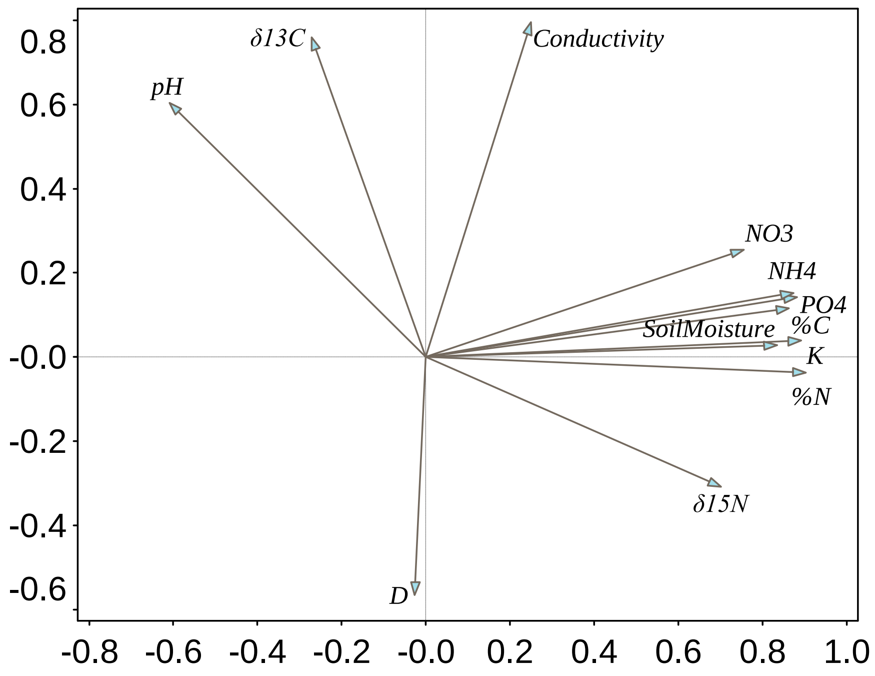

Fig. 1. Principal component analysis (PCA) plot showing relation between thirteen niche axis (presented an arrows).

Table 1. Summary of PCA axis calculated for thirteen niche axis.

Unconstrained, PCA	Total variation =1152.000			
Statistic	Axis 1	Axis 2	Axis 3	Axis 4
Eigenvalues	0.5136	0.1763	0.0793	0.0638
Explained variation	51.36	68.99	76.92	83.3

Table 2. Matrix of Spearman coefficient correlation (upper-right) and p-values (lower-left) between pairs of niche axes.

	**$\delta^{15}N$**	**$\delta^{13}C$**	**N%**	**C%**	**NH_4^+**	**NO_3^-**	**K^+**	**PO_4^{3-}**	**pH**	**Moisture**	**Cond.**	**D**
**$\delta^{15}N$**	---	-0.35	0.79	0.70	0.71	0.78	0.54	0.79	-0.63	0.42	0.12	0.18
**$\delta^{13}C$**	<0.001	---	-0.44	-0.20	-0.24	-0.31	-0.26	-0.43	0.43	-0.10	0.00	-0.08
**N%**	<0.001	<0.001	---	0.84	0.78	0.85	0.63	0.82	-0.58	0.57	0.09	0.28
**C%**	<0.001	0.052	<0.001	---	0.73	0.76	0.49	0.71	-0.40	0.49	0.34	0.13
**NH_4^+**	<0.001	0.017	<0.001	<0.001	---	0.75	0.76	0.80	-0.53	0.73	0.18	0.24
**NO_3^-**	<0.001	0.002	<0.001	<0.001	<0.001	---	0.55	0.78	-0.52	0.59	0.34	0.22
**K^+**	<0.001	0.011	<0.001	<0.001	<0.001	<0.001	---	0.69	-0.58	0.67	0.11	0.17
**PO_4^{3-}**	<0.001	<0.001	<0.001	<0.001	<0.001	<0.001	<0.001	---	-0.54	0.53	0.22	0.19
**pH**	<0.001	<0.001	<0.001	<0.001	<0.001	<0.001	<0.001	<0.001	---	-0.43	0.13	-0.20
**Moisture**	<0.001	0.313	<0.001	<0.001	<0.001	<0.001	<0.001	<0.001	<0.001	---	0.22	0.25
**Cond.**	0.236	0.996	0.398	0.001	0.074	0.001	0.295	0.033	0.192	0.030	---	-0.21
**D**	0.084	0.431	0.006	0.193	0.017	0.030	0.102	0.067	0.052	0.015	0.039	---
